# The EDLC Energy Storage Device Based on a Natural Gelatin (NG) Biopolymer: Tuning the Capacitance through Plasticizer Variation

**DOI:** 10.3390/polym14225044

**Published:** 2022-11-21

**Authors:** Shujahadeen B. Aziz, Elham M. A. Dannoun, Sozan N. Abdullah, Hewa O. Ghareeb, Ranjdar M. Abdullah, Ari A. Abdalrahman, Muaffaq M. Nofal, Sunanda Kakroo

**Affiliations:** 1 Hameed Majid Advanced Polymeric Materials Research Lab., Physics Department, College of Science, University of Sulaimani, Qlyasan Street, Kurdistan Regional Government, Sulaimani 46001, Iraq; 2 The Development Center for Research and Training (DCRT), University of Human Development, Kurdistan Region of Iraq, Sulaymaniyah 46001, Iraq; 3 Associate Chair of the Department of Mathematics and Science, Woman Campus, Prince Sultan University, P.O. Box 66833, Riyadh 11586, Saudi Arabia; 4 Department of Chemistry, College of Science, University of Sulaimani, Qlyasan Street, Kurdistan Regional Government, Sulaimani 46001, Iraq; 5 Department of Mathematics and Science, Prince Sultan University, P.O. Box 66833, Riyadh 11586, Saudi Arabia; 6 Physics Department, Faculty of Science, Jazan University, P.O. Box 114, Jazan 45142, Saudi Arabia

**Keywords:** natural polymer, energy storage device, NaNO_3_ salt, glycerol plasticizer, EIS, LSV and TNM, CV and GCD

## Abstract

A solution casting method has been utilisedto fabricate plasticisednatural gelatin (NG)-based polymer electrolyte films. The NG electrolyte with 50 wt.% glycerol and 13 wt.% sodium nitrate (NaNO_3_) attained the highest ionic conductivity of 1.67 × 10^−4^ S cm^−1^. Numerous techniques were used to characterisethe NG films to assess their electrochemical performance. The data obtained from impedance spectroscopy for the plasticisedsystem, such as bulk resistance (Rb), arerelatively low. Thiscomprehensive study has been focused on dielectric characteristics and electric modulus parameters. The plasticisedsystem has shown eligibility for practice in energy storage devices with electrochemical strength up to 2.85 V. The TNM data based on ion transference number (tion) and electron transference number (te) determine the identity of the main charge carrier, ion. The redox peaks in the cyclic voltammograms have not been observed as evidence of charge accumulation other than the Faradaic process at the electrode–electrolyte interface. The GCD plot reveals a triangle shape and records arelatively low drop voltage. The high average efficiency of 90% with low ESR has been achieved over 500 cycles, indicating compatibility between electrolyte and electrode. The average power density and energy density of the plasticisedare 700 W/kg and 8 Wh/kg, respectively.

## 1. Introduction

Electrolytes facilitate the ionic mobility between electrodes and are thus regarded as the core electrochemical devices. However, due to tsome disadvantages of liquid electrolytes, such as electrolyte leakage, high flammability and chemical instability, they are replaced by polymers [1]. On the other hand, solid polymer electrolytes (SPEs) are better in certain ways, including outstanding electrochemical stability, adaptability, high specific energy, and simplicity of fabrication into thin films [2]. Owing to all these properties, SPEs can be used in a range of solid-state electrochemical devices, such as supercapacitors, batteries, fuel cells, and chemical sensors [3]. However, low electrical conductivitymust be optimised, which is a major problem with SPEs [4]. There are frequently crystalline and amorphous phases present in polymer electrolytes’ chemical structures [5]. Based on what is known, ionic transport is exclusively available in the amorphous part of SPEs [6]. Currently, it has been demonstrated that the use of biopolymer-based electrolytes is essential for future applications in electrochemical devices such as EDLCs and batteries [6,7,8,9,10]. Gelatin, a proteinrich in proline and hydroxyproline amino acids, can be obtained by hydrolysis collagen [11]. It also originates from the bones and skins of animals. Bovine and porcine have previously been used extensively as foaming agents, emulsifiers, biodegradable packaging materials, and colloids [11,12]. Furthermore, the formation of a flexible and practically colorless film allows this biochemical substance to be frequently employed in food sector packaging. Pigskin and other sources of mammalian gelatin are costly because of the rising demand and intense manufacturing competition. Therefore, researchers have found gelatin as an appropriate substitute [13].

**Figure 1:**
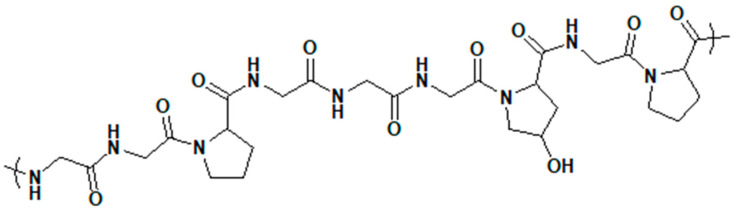
Chemical structure of Gelatin [14].

Amide and hydroxyl groups are normally abundant in the gelatin structure [14]. The presence of lone pair electrons at the heteroelements, as shownin Figure 1, is responsible for ionic conduction. The film made from gelatine doped with ionic salts, such as lithium perchlorate, lithium chloride [15], and europium triflate [16], has comparatively good transparency, adhesiveness, plasticity, and electrical current conductivity. The world has seen a progressive development of flexible energy devices, including supercapacitors, batteries, and DSSC in reference to a proper electrolyte [17,18,19].

**Figure 2:**
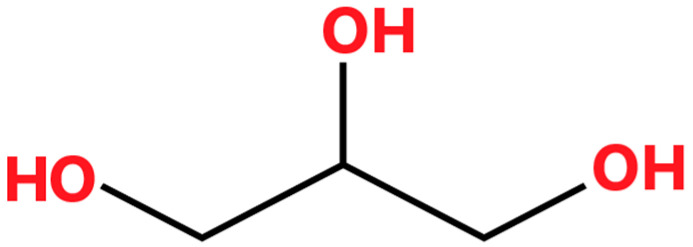
Chemical structure of glycerol.

Electrolytes play an important part in the construction of energy devices. Several strategies have been developed to increase the conductivity of the electrolyte, particularly polymer blending, salt addition, and plasticiser used. In research conducted by Chai and Isa [20], it was revealed that adding glycerol into CMC solution couldimproveionic conductivity and mechanical strength. Figure 2 shows that glycerol possesses many OHmoieties in its backbone chain, enabling ion transport in polymer electrolytes after salt addition.

The electrical double-layer capacitor (EDLC) is a reasonably simple supercapacitor device (SCD) which uses carbon electrodes since this element comesin different allotropes and possesses a large surface area, high electrical conductivity and porosity [21]. The charged ions are prominently adsorbed and desorbed onto the large surface area of carbon [22]. As it is known that the key factors in assessing EDLCs consist of specific capacitance (Cspe), equivalent series resistance (Resr), energy (E) and power density (*p*),the technology of EDLCs can find applications in electronics, communication devices, and hybrid vehicles [23]. Designing efficient EDLCs with materials that haverelatively high electrochemical capacitance remains a great challenge. Activated carbon (AC) is regarded as the optimum active component for the electrodes in EDLCs due to its large specific surface area (2500 m^2^/g), excellent conductivity, and affordable price [16]. Carbon black (CB) is the most often utilized carbon type in electrode manufacturing. The CB is a reinforcing filler used to improve dimensional stability and conductivity. One ongoing concern with CB is its smallersurface area than AC, approximately 25 to 1500 m^2^/g [24]. Condensed matter physics has several fascinating areas of study, including the enhancement of dielectric relaxation and ion conduction mechanisms in solids. Investigation of dielectric relaxation in SPEs primarily focuses on acquiring information regarding the nature of cation–polymer interactions. The dielectric constant value indicates a polymer material’s capacity to liquefy salts [25,26].

In view of all previous studies conducted on SPEs, incorporating natural beef gelatin (NBG) and sodium salts has not been dealt with yet. The key purpose of the current work is to employ a novel, inexpensive, biodegradable SPE based on NBG:NaNO_3_ doped with various amounts of plasticiseras the electrolyte. Furthermore, the impact of plasticiseramount on a SPE’s electrical and structural features has been studied.

## 2. Experimental Methodology

### 2.1. Electrolyte Preparation

A solution casting process was used to prepareseveralunplasticisedandplasticisedNBG systems. To prepare five distinct unplasticized NBG solutions, 1g of NBG was dissolved in 60 mL of 1 wt.% acetic acid. This was followed by preparing five further solutions separately by dissolving a predetermined amount of NaNO_3_ (13 wt.%) in those NBG solutions. 

The final series of NBG:NaNO_3_ solutions were subsequently plasticisedwith varying amounts of glycerol while continuously stirring until clear homogeneous solutions were obtained at room temperature. In order to enable the solvent to evaporate entirely at room temperature, each solution was individually poured onto a series of clean Petri dishes (8 cm in diameter) and enclosed with filter paper. The samples in each Petri dishes were coded as follows: BGNN0, BGNN1, BGNN2, BGNN3, BGNN4 and BGNN5 with glycerol content of 0, 10, 20, 30, 40 and 50 wt.%, respectively. Table 1 shows the NBG:NaNO_3_:glycerol film composition for each created film. 

### 2.2. Electrochemical Impedance Spectroscopy (EIS)

The NBG films weretested using impedance data at a frequency rangeof 50 Hz–MHz using the LCR meter (HIOKI 3531Z HITESTTER, Japan) connected to a computer. The real and imaginary components of impedance spectra were found by sandwiching the NBG film among two stainless steel blocking electrodes.

The electrochemical study of NBG films (i.e., BGNN5) was performed in an attempt to specify the decomposition voltage (electrochemical stability) using LSV (Digi-IVY DY2300 potentiostat, Neware, Shenzhen, China) at room temperature. The operation potential ranges from 0 to 2.5 V, with a sweep rate of 10 mV s^−1^. 

For the BGNN5 sample, the transference number (TNM) was determined using the DC polarizationmethod at a constant voltage of 0.2 V at room temperature. In the same way astheimpedance measurement procedure, the NBG film was placed between two stainless steel electrodes. The record of the current–time profile was obtained from the V&A device (Shenzhen, Neware, China, DP3003 digital DC power).

### 2.3. EDLC Fabrication

The electrode construction for EDLCs was initiated by implementing the dry mix procedure. It comprised a 0.25g carbon black (CB) and 3.25 g activated carbon (AC) in a planetary ball miller, followed by dispersing in a solution of N-methyl pyrrolidone (NMP) (15 mL) and polyvinylidene fluoride (PVdF) (0.5 g). This mixture was stirred for a few hours until the appearance of a black solution. 

Afterwards, the black solution was used to cover a current collector (i.e., Al-foil) using a doctor blade. In an oven, the collector was maintained at 60 °C to ensure the coating and drying of the film over the electrode (geometric area 2.01 cm^2^ and thickness of ~0.02 cm). Prior to use, the collector was kept in a desiccator containing silica gel.

The general cell configuration is shown below:

AC electrode|conducting SPE|AC electrode in the EDLC. 

The CR2032 coin, as an electrochemical cell, was packed with the above components. As a preliminary test for evaluating the EDLC, cyclic voltammetry was recorded.

### 2.4. CV Measurements

For the BGNN5 sample, cyclic voltammetry (CV) was conducted between potentials of 0 and 0.9V at various sweep rates. 

## 3. Results and Discussion

### 3.1. Impedance Spectroscopy Study

**Figure 3:**
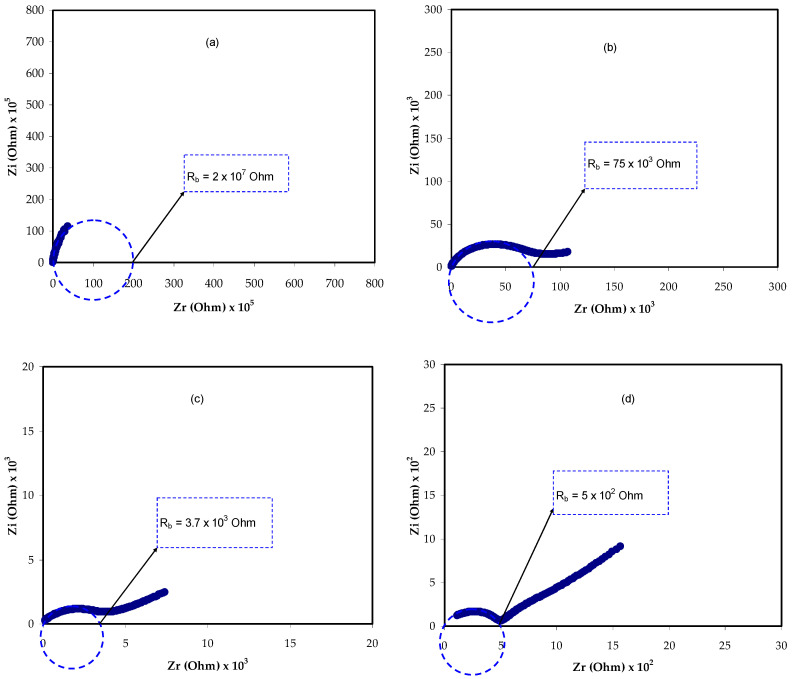

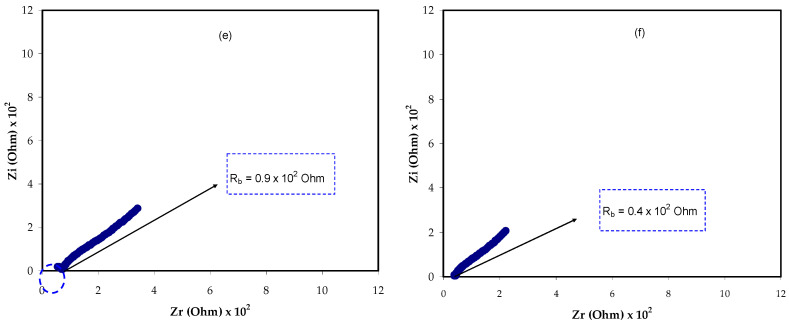
EIS plot for (**a**) BGNN0, (**b**) BGNN1, (**c**) BGNN2, (**d**) BGNN3, (**e**) BGNN4 and (**f**) BGNN5.

The impedance spectra were analyzedto investigate the ionic conductivity of polymer electrolytes. Equation (1) elucidates the established relation between ionic conductivity and the number and mobility of charge carriers [27,28].

(1) $$\sigma =\sum_{i}n_{i}q_{i}\mu_{i}$$

where $n_{i}$, $q_{i}$ and $\mu_{i}$ are mobile ion number, the charge on the mobile carrier, and the mobility of charge carrier, respectively. Equation (2) is helpful to calculate the DC ionic conductivity (σ_dc_) of NBG electrolyte films.

(2) $$\sigma_{dc}=\frac{l}{R_{b}A}$$

where l, $R_{b}$, and A represent film thickness, bulk resistance and surface area of electrolyte film, respectively. In Figure 3, the impedance spectra for all samples are exhibited. The impedance spectra can be recognisedfrom two features; a high-frequency semicircle and a low-frequency spike (straight line). The reason forthe appearance of spike response is the free charge accumulation at the interfaces between the solid electrolyte and the electrode surface. This causes the formation of an electric double-layer capacitor [22]. The polymer electrolytes have bulk conductivity, which isresponsible for the semicircle response [23].

In Figure 3b–e, the presence of the low-frequency spike and the diameter reduction inthe high-frequency semicircle can be attributed to blocking ion transport at stainless-steel electrodes, i.e., hindering double-layer capacitance at the blocking electrodes [24]. Furthermore, with 20–40% plasticiseraddition, the ascending semicircle gradually shrinksat the high-frequency region. This occurs due to a drop in the NBG electrolyte films’ bulk resistance (R_b_) and, in turn, an increase in ionic conductivity [25]. It is apparent from Figure 3f that the semicircular portion has vanished. An explanation for this is the existence of glycerol as a plasticiserfacilitating ion migration through increased flexibility. The precise value of R_b_ can be determined from the data analysis by taking the straight line’s intercept on the real axis of the impedance plot. In Table 2, the σ_dc_ is quantified for all NBG electrolyte films. The addition of plasticiserimproved the ionic conductivity from 10^−10^ to 10^−4^ S/cm.

Through the inhibition of ionic crystal growth, the plasticisercan also contribute to improving ionic conductivity, meaning that it reduces the columbic interaction. Collectively, salt and plasticiserenhanceionic conductivity increase the $n_{i}$ and $\mu_{i}$, respectively [29]. In this study, the highest σ_dc_ measured was 1.67 × 10^−4^ S cm^−1^.

### 3.2. Dielectric Properties

**Figure 4:**
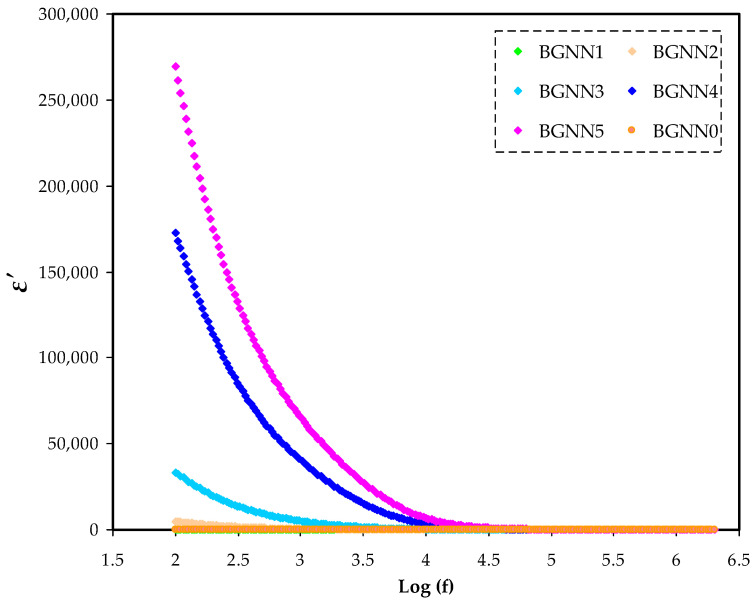
The ɛ′ spectra for the NBG electrolyte systems.

**Figure 5:**
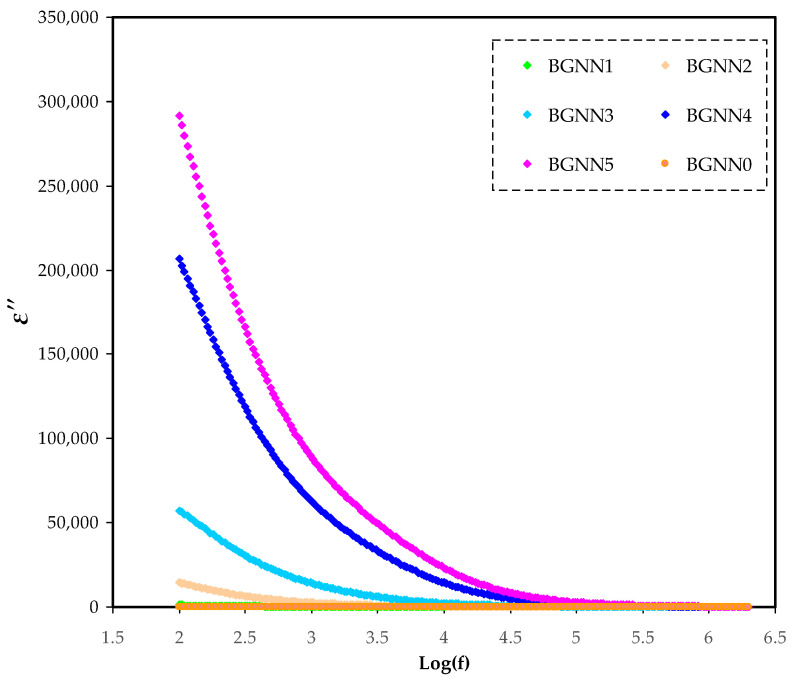
ɛ″ spectra for the NBG electrolyte systems.

In Figure 4 and Figure 5, the frequency dependence of dielectric constant (ɛ′ or ɛ_r_) and dielectric loss (ɛ″ or ɛ_i_), respectively, is represented. Obviously, as the frequency increases, both ɛ′ and ɛ″ decrease proportionally to the minimum, followed by a plateau at high frequencies. The high values of ɛ′ and ɛ″ at low frequencies can be explained based on electrode polarization(EP) [30,31], which is the resultant of charge accumulation at the electrode–electrolyte interface [32]. In the ɛ″ spectra, the lack of dielectric relaxation peaks is a consequence of masking during the segmental relaxation behaviour of the polymer by σ_dc_ of carriers [30,33]. The loss peaks in polymer electrolytes arehidden because of the masking of dipolepolarizationrelaxation by mobile charged species at high electrical conductivity [34].

**Figure 6:**
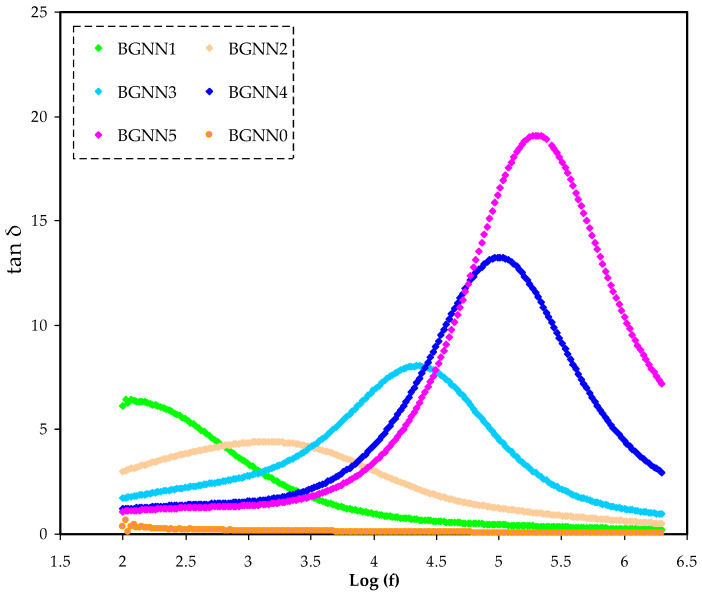
Loss tangent (tan δ) spectra for the NBG systems.

To comprehensively understand the relaxation process, the dielectric loss tangent (tanδ) versus frequency is the best choice to focus on, as shown in Figure 6. It is seen that there is a peak that resulted from the translational ion dynamics, reflecting the conductivity relaxation of mobile ions [35,36]. The phenomenon within the polymer electrolyte is the polymer segmental mobility that shortens the relaxation time and increases the charge transferring. In the relationship of τ = 1/2π*f*_max_, τ is the relaxation time for the ionic carriers [37]. Thus, the higher plasticisercontent in the polymer electrolyte can improve the flexibility of segmental motion as well as ionic transport. The tanδ plot’s response shape is built upon Koop’s phenomenological principle [38,39]. 

Hence, tanδ increases with frequency until it reaches its maximum value at varyingtemperatures, after which it decreases. This is because the low-frequency pattern raises the ohmic component of current more significantly than the capacitive one. In contrast, the high-frequency pattern corresponds to rising the capacitive component in response to frequency, but the ohmic component of the current remains nearly unchanged with frequency [38,40]. The final feature observed in the tanδ plot is the broad peak, identifying the non-Debye type of the relaxation process [41]. The plot also shows that when the plasticizer increases, the tanδ peak maximum shifts to a higher frequency. As the plasticizer increases, the peak frequencies shift forward, implying that the relaxation time decreases (see Table 3). As abovementioned, this behaviour leads to the fact that a parallel RC element can express the system. According to (*σ_dc_ = L/R_b_A*), R is proportional to conductivity. From this relation *σ = σ_o_exp(E_a_/KTɛ′)*, where E_a_ is the activation energy, σ_dc_ is increased with an increase in the plasticizer content and thus the (tanδ)_max_ shifts to the higher frequencies owing to ɛ′ improvement of the electrolyte system by plasticisersɛ′ value [42]. The broadness of the tanδ peaks shows the existence of more than one relaxation process, which is non-Debye type one [41]. 

### 3.3. Electric Modulus Analysis

**Figure 7:**
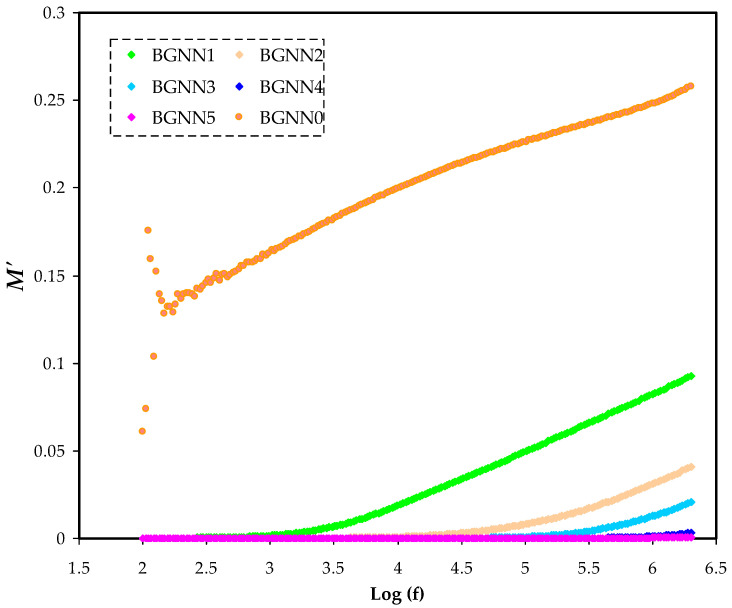
The M′ spectra for the NBG electrolyte systems.

**Figure 8:**
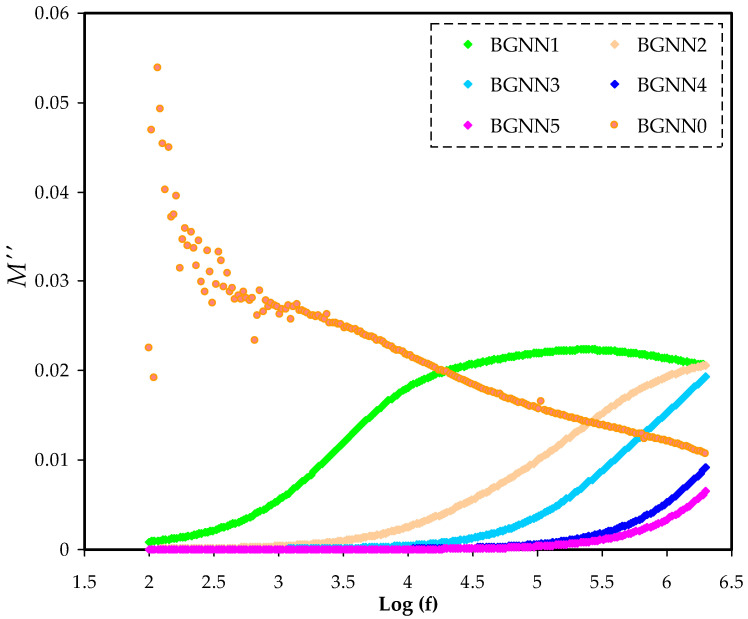
The M″ spectra for the NBG electrolyte systems.

A complex phenomenon in polymer electrolytes is ion conductivity. Studying a polymer’s dielectriccharacterstics induced by ion response involves using electric modulus parameters, represented by the reciprocal electric permittivity. In polymer electrolytes, examination of this modulus can be used to control the EP, which acts to suppress the charge accumulation near the electrodes [6,43,44,45]. Equations (3) and (4) illustrate how dielectric modulus assessment is used to obtain a deep knowledge of the dielectric permittivity of the systems [6,45].

(3) $$M^{\prime }=Z_{i}C_{o}\omega$$

(4) $$M^{\prime \prime }=Z_{r}C_{o}\omega$$

where the actual and the pretend components of electrical modulus are symbolised by $M^{\prime }$ and $M^{\prime \prime }$,respectively [46]. For all NBG samples at room temperature, the values of each component as a function of frequency are presented in Figure 7 and Figure 8, respectively.At the stumpy frequency region, a characteristic long tail is caused by the polarizationphenomenon providing a high capacitance related to electrodes and a high ɛ′. At the elevated frequency region, the values of $M^{\prime }$ and $M^{\prime \prime }$ were visible and certain relaxation peaks were seen as a consequence of bulk modulus formalism effects. This mostly indicates the ionic conductivity of the NBG electrolyte films [45]. In addition, once the plasticiserwas added to the polymer electrolyte film (BGNN5), these relaxation peaks were deformed at the high-frequency regions. This can also be explained by involving numerous ions to enhance conductivity. Interestingly, the greaterthe amountof glycerol, the greater the mobility of charge transport ions.From impedance plots, it was found that the BGNN1 sample is too insulative. The scattering observed in the BGNN1 spectrum in Figure 7 and Figure 8 may be attributed to the low conductivity behaviour of the sample.

### 3.4. AC Conductivity Analysis (σ_AC_)

**Figure 9:**
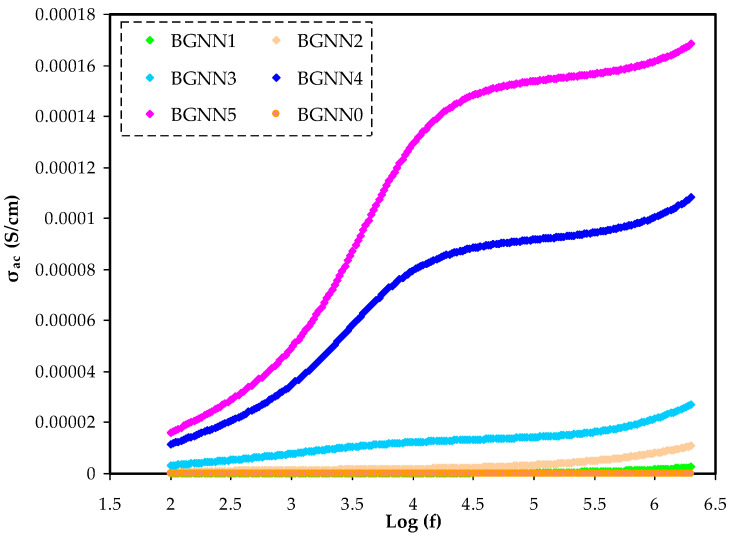
The σ_ac_ spectra for the NBG electrolyte systems.

Figure 9 displays the AC conductivity (σ_AC_) trends for the unplasticisedandplasticisedNBG samples as a function of the applied electric field frequency. To determine σ_AC_ for all these film systems, the following equation is used [47]:

(5) $$\sigma_{AC}=\left[\frac{Z^{\prime }}{{Z^{\prime }}^{2}+{Z^{\prime \prime }}^{2}}\right]\times \frac{d}{A_{}}$$

It is imperative to notice that the electrical conductivity behaviour of the NBG films in the frequency dependence of the dispersion region obeys Jonscher’spower law as expressed by [48,49]:

(6) $$\sigma_{\left(\omega \right)}=\sigma_{DC}+A\omega^{n}$$

In this equation, $\sigma_{\left(\omega \right)}$ represents the overall conductivity resulting from AC and DC and $\sigma_{DC}$ denotes the frequency-independent conductivity. Other variables include the temperature and sample composition dependence of the parameter A and the frequency exponent n that correlates with the hopping rate to the relaxation time of site groups and takes the values from 0 to 1 [50]. It is evidentthat the σ_AC_ increases with frequency. The reason is that under the influence of the applied electrical signal, the charge carriers are excited, increasing their mobility, reducing the relaxation period and raising conductivity [37]. A precise prediction of σ_DC_ is by using the frequency of the applied electrical signal as a measure of σ_AC_ [51,52,53]. For materials with considerable σ_DC_, three distinct regions are recognisedfrom their σ_AC_ spectra [54], as exemplified in Figure 9. The low-frequency data are driven by the EP, whereas the intermediate region data are driven by σ_DC_. Based on a previous study, the conductivity spectrum’s divergence from the DC value (the plateau region) is caused by the impact of EP [53].

### 3.5. EDLC Study

#### 3.5.1. TNM and LSV Analysis

**Figure 10:**
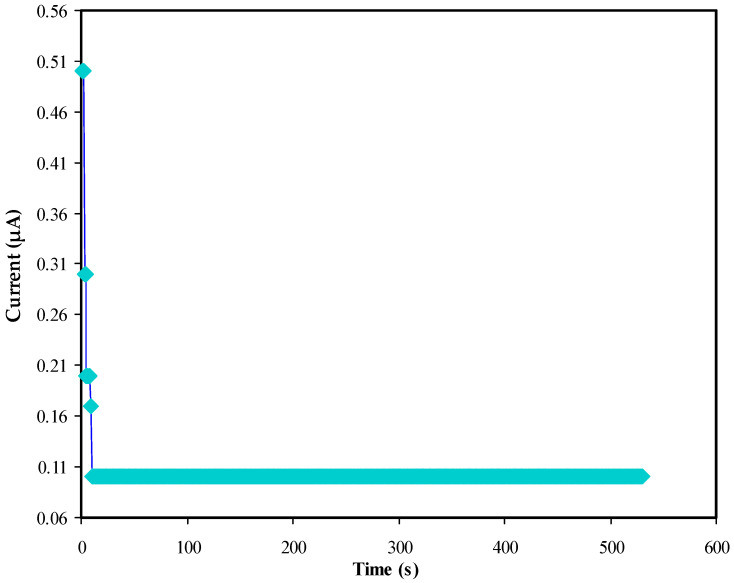
Polarization current vs. time for the BGNN5 system (t_ion_ = 0.8 and t_e_ = 0.2).

The usefulness of the present NBG electrolyte system for EDLC applications can be tested based on the *t_i_* value, which must be high enough. Ion transport (*t_ion_*) and electron transport (*t_e_*) are crucial parameters to consider when assessing the conductivity of electrolyte materials. The transfer number measurement (TNM) involves ion/electron transfer, its non-blocking electrode system is designed to allow both ions and electrons to pass, and its blocking electrode system blocks the ions while enablingelectron transport. The ideal target in the polymer electrolytes is the greater value of *t_ion_* than *t_e_* [55,56]. Figure 10 shows the TNM plot of the BGNN5 system. As can be seen, a current value of 0.5µA is initially recorded, followed by a sharp decline until 15 s. This current value is relatively high enough. After 20 s, the current reaches a steady state recorded at 0.1 µA. The sharp decline in the current can be interpreted as evidence of transporting charge by electron via stainless steel. In this research, the record of *t_i_* for the conducting NBG:NaNO_3_:glycerol system is set up to be 0.8. In general, the ions are primarily responsible for charge transport in the present polymer electrolyte.

**Figure 11:**
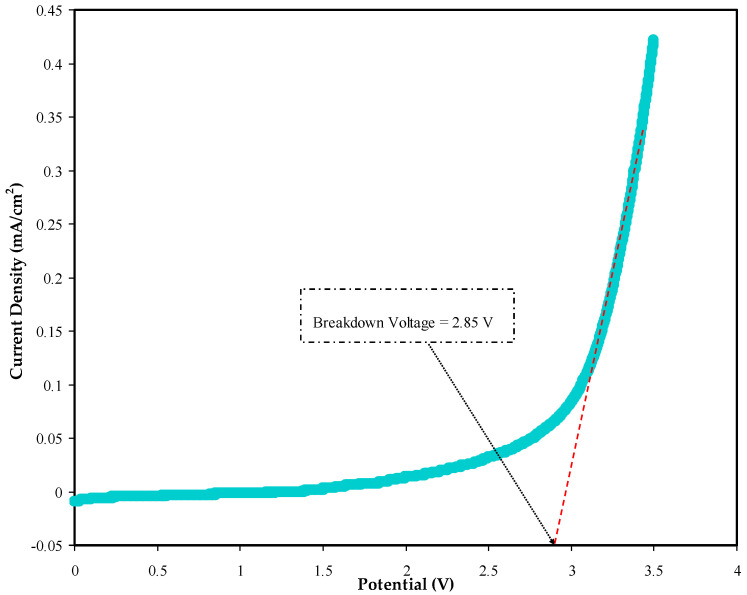
Determination of decomposition voltage for the BGNN5 system with the highest conductivity through the LSV plot.

**Figure 12:**
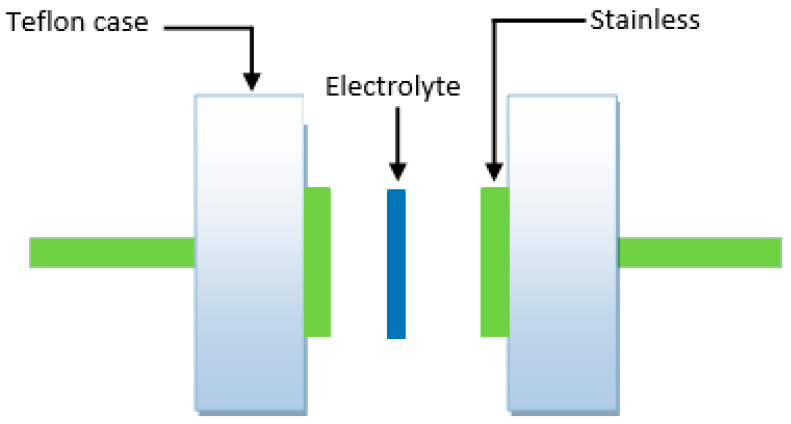
Schematic representation for recording TNM and LSV data.

Another important criterion in determining the application of an electrolyte is its stability against decomposition following the current passage [57,58,59]. LSV data can provide information on the electrochemical stability of polymer electrolytes(see Figure 11), and the cell design is shown in Figure 12 illustrates the determination of the potential window for the plasticized BGNN5 system.

Yusof et al. reported the potential stability of a biopolymer-glycerol system at a voltage < 1.9 V [60]. In accordance with this value, the present electrolyte can be used as an electrode separator within energy devices. Above 2.85 V, the polymer electrolyte begins to decompose, resulting in a dramatic rise in current caused by the electrolyte’s breakdown at the inert electrode surface. It has been demonstrated that a polymer electrolyte can be used in energy devices if the operating voltage is close to 1.0 V [61].

#### 3.5.2. Cyclic Voltammetry Study (CV)

**Figure 13:**
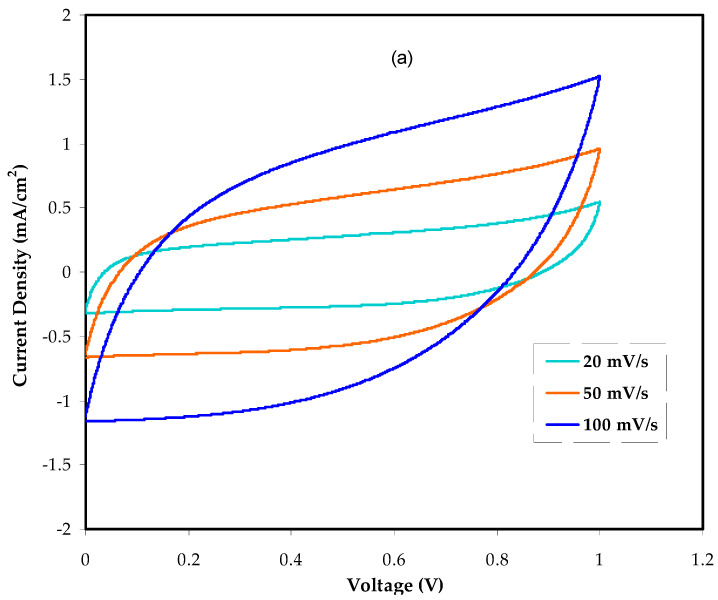

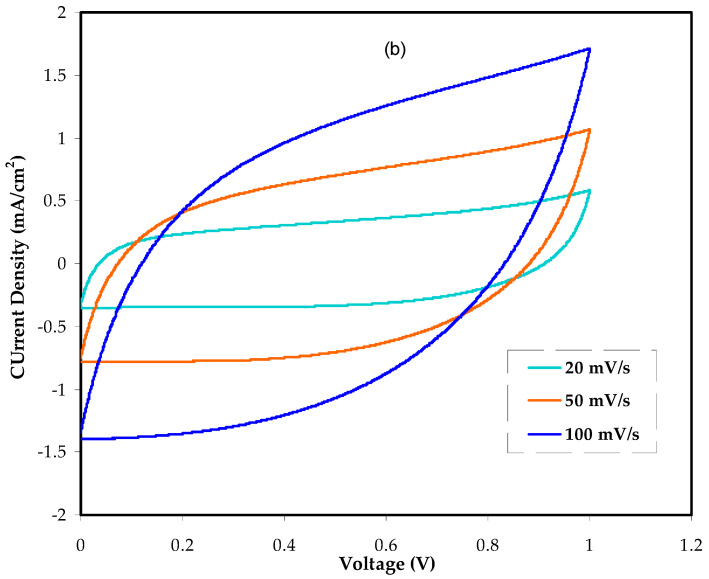
The CV patterns of the designed EDLC for the (**a**) BGNN5 and (**b**) BGNN4 systems.

The electrolyte’s cyclic voltammetry can be recorded to understand Faradaic and non-Faradaic processes fully. The charge storage at each interface in anodic and cathodic regions of the EDLC is accomplished via a non-Faradaic process [62,63,64]. Figure 13a,b show the CV profiles obtained for BGNN5 and BGNN4 systems at different scan rates, respectively. The fundamental characteristic of the CV profile is often its rectangular form and lack of redox peaks. At 100 mV/s, the CV is expected to have a leaf-shaped profile with a broad area. This is because the non-Faradaic process dominates the Faradaic one. Based on the CV profiles at different scan rates, the specific capacitance (*C_s_*) of the EDLC can be computedas shown in Equation (7):

(7) $$C_{s}=\int_{V_{i}}^{V_{f}}\frac{I\left(V\right)dV}{2mv\left(V_{f}-V_{i}\right)}$$

**Figure 14:**
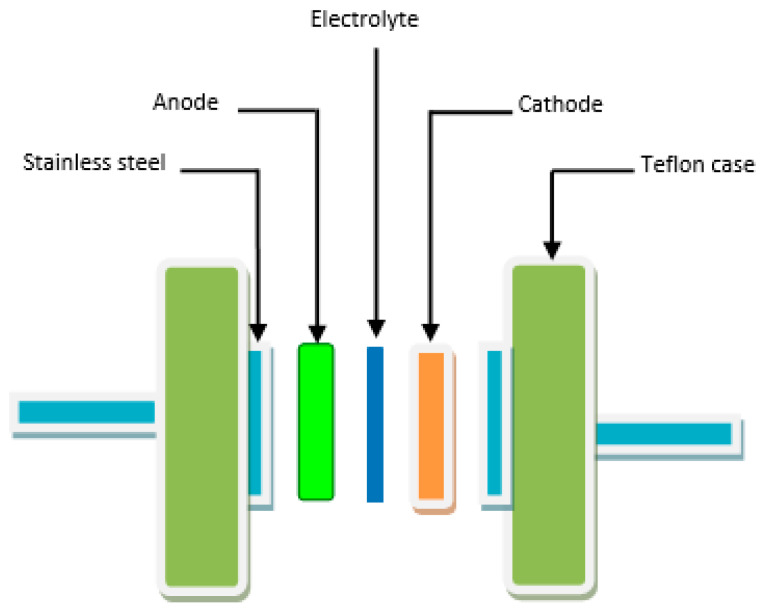
Schematic representation of the fabricated EDLC cell.

Here, $V_{i\;}$ and $V_{f}$ are the initial and final voltages, respectively; m is the mass of active material, v is the scan rate and ʃ(V) dV is the region underneath the CV loop. Figure 14 shows the schematic representation of the fabricated EDLC cell. 

In Table 4, the cell *C_s_* at various scan rates is listed. With an increase in scan rate, the *C_s_* values seem to decrease correspondingly. This occurs because the extended charge diffusion duration cannot track the variations of electric field and high power density at high scan rates. Furthermore, there are interrelations between the *C_s_* value and *t_ion_* resistance, diffusion speed and diffusion length [65,66]. 

At 100 mV/s, the *C_s_* value for the BGNN4 and BGNN5 systems is comparatively low (5.97 and 6.95 F/g, respectively). The high scan rate causes ions in the electrolyte to move very quickly toward the electrode surface, hindering the development of efficient double-layer formation and resulting in low *C_s_*. However, at 20 mV/s, the CV responses are nearly rectangular, recording a*C_s_* of 9.77 and 11.90 F/g for theBGNN4 and BGNN5 systems, respectively. A high *C_s_* can therefore be accomplished at stumpy scan rates as the ions pile up mainly at the interfacial region, which causes the electrode surface to be appropriately polarized [67]. According to Pal et al.’s study, an ideal rectangular CV is unachievable because of both internal resistance and activated carbon porosity [22]. Remarkably, there is no peak at either the low scan rate (20 mV/s) or the higher one (100 mV/s). To alarge extent, the results of the present system are unfailing with the energy storage mechanism of a capacitor. The creation of double-layer charge at the interfacial region is originated from the electron distribution at the electrode and ion accumulation from the electrolyte.

#### 3.5.3. Galvanostatic Charge Discharge (GCD) Study

**Figure 15:**
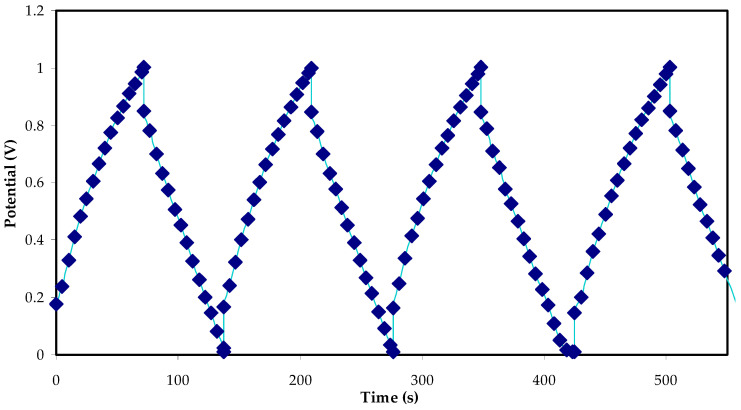
The GCD side view of the EDLC.

In the GCD, the implemented potential range is 0.0–0.9 V. In Figure 15, the charge–discharge profile of the fabricated EDLC device is shown. The internal resistance in the EDLC is evidenced bythe electrolyte *R_b_* and the gap between the electrolyte and the current collectors. The existence of internal resistance by reducing the potential before charging and discharging is confirmed. Furthermore, the linear slope of the EDLC indicates its utility as a requisite energy storage capacitor [68]. There is also a potential decline beforethestartof the discharge process. As previously mentioned, the factors contributing to this include the electrolyte *R_b_* and the distance between the electrolyte and the current collectors. 

**Figure 16:**
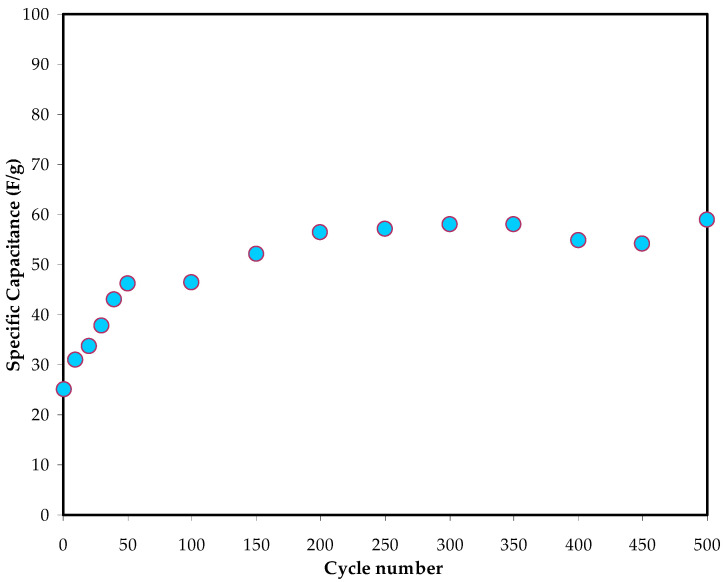
Variation in *C_s_* value during 500 cycles.

Clearly, it is understood that the virtually linear discharge slope of the charge–discharge profile proves the capacitive behaviour in the EDLC [27]. From this profile for 500 cycles, the *C_s_* values of the EDLC can be derived using Equation (7), and the resulting values are presented in Figure 16. At the 1st cycle, the *C_s_* value is equal to 25 F/g. Interestingly, the values of *C_s_* obtained from the charge–discharge profile, and the CV differ slightly. Nevertheless, this slight difference in *C_s_* values is acceptable, indicating the reliability of these results as an EDLC capacitor cell [60]. 

**Figure 17:**
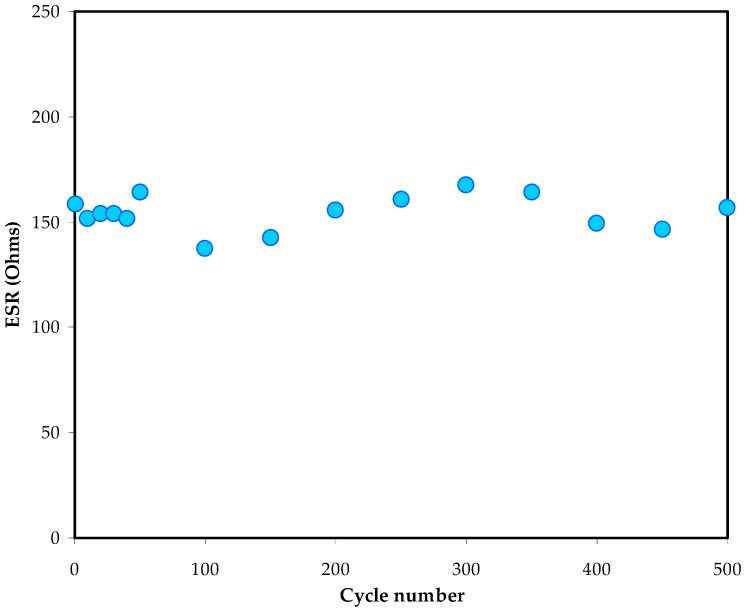
ESR of the EDLC for 500 cycles.

In Figure 17, the R_ESR_ inclination of the EDLC for 500 cycles of charging and discharging is shown. Low ESR values imply that the electrolyte|electrode contact in the EDLC assembly is compatible. This reveals that the ions are conveniently moved from the electrolyte’s bulk region to the surface of activated carbon, creating a charge space double-layer with low internal resistance [69]. 

During the cycling stability of the system, the coulombic efficiency (*η*) of the EDLC assembly has to be determined using the following relation:

(8) $$\eta =\frac{t_{d}}{t_{c}}\times 100\;\;\;\;\;\;\;\;$$

**Figure 18:**
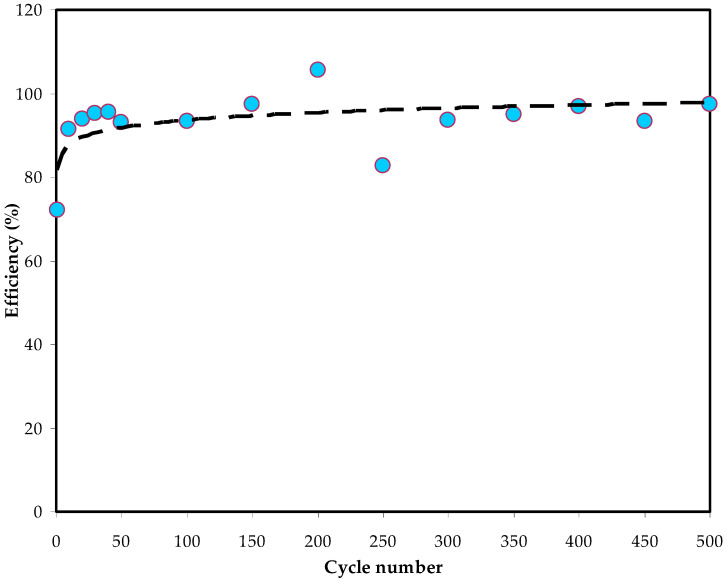
Efficiency prototype of the EDLC device over 500 cycles.

Here, *t_c_* and *t_d_* are the period of charge and discharge processes, respectively. Figure 18 displays the efficiency of the studied EDLC for 500 cycles. Resultsof 74% and 92% are achieved at the 1st cycle and 110th cycle, respectively. From the 300th cycle onwards, efficiency plateaus and maintains its value at approximately 90%. According to Lim et al. [70], electrode–electrolyte contact in the EDLC is feasible if the efficiency exceeds 90.0%. At the initial stage, charging takes longer than discharging [71]. Once the voltage is supplied, the conduction begins by directing ions toward the electrode surface, resulting in low efficiency. Before the 5th cycle, efficiency remains almost constant at 95% and fluctuates between 97 and 99% until the 500th cycle. The duration of charging and discharging isalmost similar at the high-efficiency value. A good contact in the EDLC assembly and a reliable double-layer structure contribute to minimizing the charge loss up to 500 cycles.

Evaluation of the EDLC assembly via calculating the efficiency of the system can be achieved from both *E* and *p* using the followingequations:

(9) $$E=\frac{C_{s}V^{2}}{2}$$

(10) $$P=\frac{V^{2}}{4m\left(ESR\right)}$$

**Figure 19:**
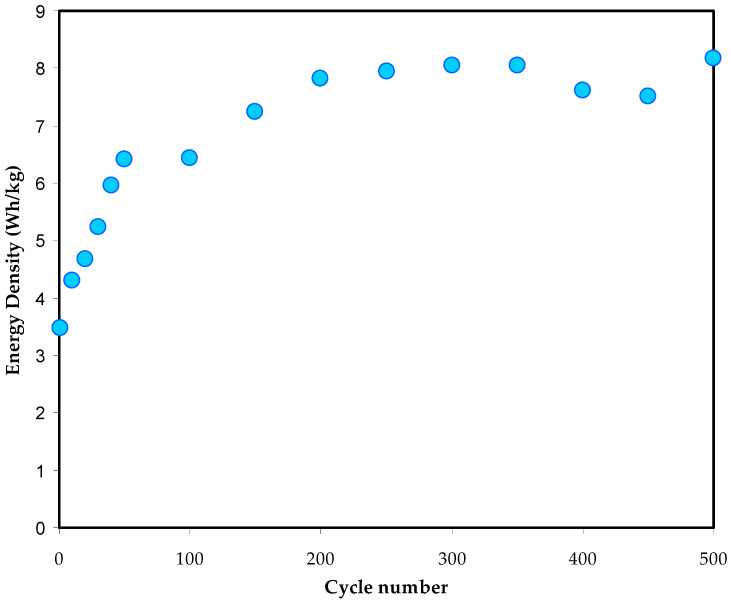
Energy density (*E*) trend of the fabricated EDLC over 500 cycles.

Figure 19 illustrates the *E* values of the EDLC assembly calculated over 500 cycles. At the 1st cycle and 90th cycle, the *E* resultsare steeply increased from 3.5 to 6.5 Wh/kg, respectively. From the 100th cycle to the completion of cycling, the *E* value is consistently maintained at 8 Wh/kg.

It is critical to note that the *E* and *C_s_* values are in good agreement, as shown in Figure 19. The results indicate that the energy of charge carriers requiredfor migration towards the electrode surfaces is evenly distributed throughout the charge–discharge process [72]. 

**Figure 20:**
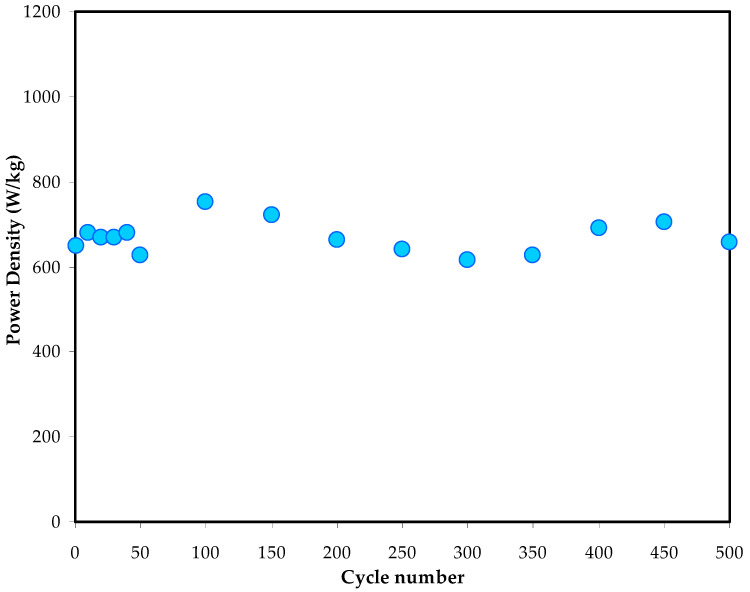
The *p* trend of the EDLC assembly over 500 cycles.

Figure 20 represents the *p* values of the EDLC assembly calculated over 500 cycles. Inthe 1st cycle, the *p* rate is 650 W/kg.Aslightchange until the end of cycling (500 cycles) can be observed. A harmonized *p* trend with the *ESR* plot is obtained. This is due to the same effect of internal resistance, which diminishes the electrolyte and promotes ion recombination. At high cycle numbers, the ion recombination can result from the fast charging and discharging mechanisms, thus reducing the *p* values [73]. In comparison, the current system possesses power and energy densities much higher than those published in the literature for polymer-based electrolytes, namely, natural and synthetic polymers.

In all types of electrochemical devices, such as lithium-ion batteries, electrochemical double-layer capacitors, quantum dot-sensitized solar cells, dye-sensitized solar cells, fuel cells and electrochromic devices, electrolytes are regarded as the core electrochemical devices [74,75,76].

## 4. Conclusions

The NBG:NaNO_3_:glycerol polymer electrolyte has been prepared byimplementing the solution casting technique. Reductions in bulk resistance with an increase inplasticiserwere observed. The boost in dielectric constant value with a rise inplasticiserisevidenceof an increase in free ions, thus increasing the system’s capacitance. The decline inrelaxation time with plasticiser reveals the improvement of ion mobility through increased flexibility of the polymer. The relaxation process associated with ions indicates a coupling among ion/polymer chain motions. Improvement of the ionic conductivity of the polymer electrolyte can be reached by enhancing ion mobility and salt dissociation. Maximum conductivity can be obtained, and the relaxation process has been identified as a non-Debye type one. Three distinguished regions characterise the AC conductivity plot: electrode, DC contribution and ac power law region. 

The electrolyte possesses a large decomposition voltage and a high ionic transference number. Descriptively, the electrolyte under study is eligible for utilization ata large scale based on the above results. The almost rectangular shape of CV reveals charge storage at the electrode–electrolyte interface as the non-Faradaic process. It seems that byusingaplasticiser, tuning the capacitance of the NBG:NaNO_3_:glycerol polymer electrolyte system can be achieved. The GCD plot reveals a triangle shape and recordsarelatively low drop voltage. The high average efficiency of 90% with low ESR has been achieved over 500 cycles, indicating compatibility between electrolyte and electrode. The average power density and energy density of the plasticisedare 700 W/kg and 8 Wh/kg, respectively.

## Figures and Tables

**Table 1:** Composition of unplasticisedandplasticisedNBG:NaNO_3_:glycerol films.

Sample Code	NBG (g)	NaNO3 (wt.%)	Glycerol (wt.%)
BGNN0	1	13	0
BGNN1	1	13	10
BGNN2	1	13	20
BGNN3	1	13	30
BGNN4	1	13	40
BGNN5	1	13	50

**Table 2:** Values of DC conductivity for all NBG electrolytes.

System	DC Conductivity (S/cm)
BGNN0	3.34 × 10^−10^
BGNN1	8.92 × 10^−8^
BGNN2	1.81 × 10^−6^
BGNN3	1.34 × 10^−5^
BGNN4	7.43 × 10^−5^
BGNN5	1.67 × 10^−4^

**Table 3:** Variation of relaxation frequency (f_max_) with relaxation time (τ).

f_max_ (Hz)	Relaxation Time (s)
122	1.31 × 10^−3^
1600	9.95 × 10^−5^
23,204	6.86 × 10^−6^
102,499	1.55 × 10^−6^
205,019	7.77 × 10^−7^

**Table 4:** *C_s_* at differentscan rates for the BGNN4 system (40 wt.% glycerol) and the BGNN5 system (50 wt.% glycerol).

Scan Rate (V/s)	*C_s_* at 40 wt.% Glycerol	*C_s_* at 50 wt.% Glycerol
0.1	5.97	6.95
0.05	7.76	9.30
0.02	9.77	11.90

## Data Availability

Not applicable.
